# Emotion Dysregulation and Eating Disorder Symptoms: Examining Distinct Associations and Interactions in Adolescents

**DOI:** 10.1007/s10802-022-00898-1

**Published:** 2022-01-14

**Authors:** Nora Trompeter, Kay Bussey, Miriam K. Forbes, Phillipa Hay, Mandy Goldstein, Christopher Thornton, Christopher Basten, Gabriella Heruc, Marion Roberts, Susan Byrne, Scott Griffiths, Alexandra Lonergan, Deborah Mitchison

**Affiliations:** 1 Centre for Emotional Health, Department of Psychology, Macquarie University, Sydney, Australia; 2 School of Medicine, Translational Health Research Institute, Western Sydney University, Camden and Campbelltown Hospital, SWSLHD, Sydney, Campbelltown Australia; 3 The Redleaf Practice, Private Practice, Sydney, Australia; 4 School of Population Health, Faculty of Medical & Health Sciences, University of Auckland, Auckland, New Zealand; 5 School of Psychological Sciences, University of Western Australia, Perth, Australia; 6 Melbourne School of Psychological Sciences, University of Melbourne, Melbourne, VIC Australia

**Keywords:** Emotion dysregulation, Weight and shape concerns, Eating disorders, Disordered eating, Adolescence

## Abstract

Emotion dysregulation has been posited as a key transdiagnostic factor of mental health difficulties, including eating disorders. However, how this transdiagnostic factor interacts with the disorder-specific factor of weight and shape concerns remains unclear. The current study examined whether emotion dysregulation is associated with eating disorder behaviors over and above the association between weight and shape concerns and whether these two factors interacted. The current study used data from two samples, a community sample of high school students (*n* = 2699), and a clinical sample of adolescents receiving outpatient treatment for an eating disorder (*n* = 149). Participants completed self-report measures on their eating behaviors, weight/shape concerns, and emotion dysregulation. Findings showed that emotion dysregulation had a unique association with engaging in binge eating and purging (community sample only). Weight and shape concerns were found to have a unique association with engaging in binge eating, fasting, purging, and driven exercise (community sample only). Additionally, weight and shape concerns moderated the association between emotion dysregulation and the probability of engaging in binge eating and driven exercise, whereby the strongest association between emotion dysregulation and these behaviors were observed among adolescents with the lowest levels of weight and shape concerns. Regarding the frequency of eating disorder behaviors, emotion dysregulation had a unique association with severity of binge eating and fasting. Weight and shape concerns were uniquely associated with severity of fasting and driven exercise (community sample only). Findings suggest that emotion dysregulation is a distinct factor of eating disorder behaviors among adolescents.

**Supplementary Information:**

The online version contains supplementary material available at 10.1007/s10802-022-00898-1.

Eating disorders are characterized by disturbances in both body image and eating behaviors (American Psychiatric Association, 2013) and typically develop during adolescence (Hudson et al., 2007; Nagl et al., 2016). These disorders are serious mental health conditions linked to reduced quality of life, psychological distress, and high co-morbidity with other mental health disorders (Ágh et al., 2016; Mitchison et al., 2015; Rojo-Moreno et al., 2015). Importantly, early detection and intervention are crucial in reducing the protracted course of these disorders and their fiscal and other costs to the individual and the community (Le et al., 2017; Treasure & Russell, 2011). As such, identifying factors associated with eating disorder behaviors in adolescents are important in informing more effective prevention and intervention programs.

Recent research has pointed to emotion dysregulation as a key factor associated with eating disorder behaviors (see Trompeter et al., 2021a, 2021b for review). Emotion dysregulation refers to problems in identifying emotions or using adaptive regulatory strategies (Gratz & Roemer, 2008; Keenan, 2000) that impact interpersonal function and personality development. Numerous studies have shown that adolescents with eating disorders have higher levels of emotion dysregulation than their peers without an eating disorder (Anderson et al., 2018; Weinbach et al., 2018), indicating a co-occurrence of emotion dysregulation and eating disorder behaviors during adolescence. Furthermore, emotion dysregulation has been linked to higher rates of disordered eating behaviors, such as binge eating and dietary restraint, both in clinical and community samples (Burton & Abbott, 2019; Goodwin et al., 2014). Thus, there is sufficient evidence to suggest that emotion dysregulation is associated concurrently with eating disorder behaviors, which is in line with theoretical models of eating disorders.

The transdiagnostic model for eating disorders ("CBT-E model"; (Fairburn et al., 2003) states that while all eating disorder behaviors share the same central underlying mechanism, overvaluation of weight/shape and eating, there are additional maintenance mechanisms involved in specific eating disorder behaviors. One of these is mood intolerance defined as “an inability to cope appropriately with certain emotional states” (Fairburn et al., 2003, p. 157), which is proposed as a maintenance factor for binge eating and purging specifically. However, we have previously argued that the model should capture not only mood intolerance, but emotion dysregulation more broadly, and pathways should also connect to other behaviors including fasting and driven exercise (Trompeter et al., 2021a). Such approaches are in line with more recent treatment models, including Integrative Cognitive-Affective Therapy (Wonderlich et al., 2008), whereby emotion dysregulation is posited as a key aspect of eating disorder behaviors. As such, the current study aimed to examine whether emotion dysregulation is uniquely associated with various eating disorder behaviors.

Further, we have proposed that research should examine the interplay between emotion dysregulation (a transdiagnostic factor) and weight and shape concerns (a disorder-specific factor) when investigating eating disorder behaviors among adolescents (Trompeter et al., 2021a). Examining the interplay between transdiagnostic factors (e.g., emotion dysregulation) and disorder-specific factors (e.g., weight/shape concerns) may provide important information about the relative importance of each factor. For example, a study by Racine and Martin (2016) found that among adult women the transdiagnostic factor of negative urgency, the tendency to act impulsively when distressed (a concept closely related to emotion dysregulation; Juarascio et al., 2020), interacted with the disorder-specific factor of body dissatisfaction. Specifically, the association between negative urgency and disordered eating was stronger at higher levels of body dissatisfaction. However, no study to date has examined such an interactive link for emotion dysregulation and weight and shape concerns. Dimensional frameworks of psychopathology suggest that symptoms manifest based on a combination of both broad risk factors and narrower syndrome-specific moderators (Forbes et al., 2016). As such, in addition to examining whether emotion dysregulation is uniquely associated with eating disorder behaviors, we also seek in the current study to examine whether high levels of weight and shape concerns strengthen these associations.

Most previous research has focused on adult women. However, given that eating disorders typically develop during adolescence (Nagl et al., 2016), examining eating disorder symptoms and their correlates during adolescence is crucial. Additionally, adolescence marks a key developmental period in terms of the onset of weight and shape concerns (Gowers & Shore, 2001), as well as emotion dysregulation (Bailen et al., 2018; Compas et al., 2017). Previous studies among adolescents support research from adult samples, showing that emotion dysregulation is linked with eating disorder behaviors (Hansson et al., 2017; McLaughlin et al., 2011). However, such studies have mainly relied on community samples of adolescents with limited research investigating this relationship in clinical samples of adolescents. Importantly, previous research has demonstrated substantive differences between community and clinical groups with disordered eating for severity of weight and shape concerns, as well as frequency of eating disorder behaviors (Trompeter, et al., 2021b). Further, the association between emotion dysregulation and mental health symptoms more broadly has been found to be stronger in clinical as opposed to community samples (Aldao et al., 2010).

To address this gap in the literature, the current study aimed to examine whether emotion dysregulation was associated with eating disorder behaviors over and above the association between weight and shape concerns, in both a community and clinical sample of adolescents. Specifically, we hypothesized, based on prior research (Burton & Abbott, 2019; Goodwin et al., 2014; Laghi et al., 2018; Lavender et al., 2014), that emotion dysregulation would be associated with both a higher probability of engaging in and more severe levels (i.e., greater frequency) of specific eating disorder behaviors (fasting, binge eating, purging, and excessive exercise). Moreover, we hypothesized weight and shape concerns would moderate the association between emotion dysregulation and eating disorder behavior, whereby the associations would be stronger at higher levels of weight and shape concerns. Given the novelty of the current study, no a priori hypotheses were made about potential differences between the community and clinical samples.

## Method

### Participants and procedure

Data were used from two samples: (1) a community sample of high school students, and (2) a clinical sample of adolescents receiving outpatient treatment for an eating disorder. The sampling procedures for each sample are outlined below. Ethical approval for both studies (the EveryBODY study and the TrEAT study) was granted by Macquarie University. Additionally, the EverBODY study was reviewed and received ethical approval from the New South Wales State Education Research Applications Process.

#### Community Sample

Participants from the community sample were part of the second wave of the EveryBODY study, a cohort study of eating disorders and body image concerns among 5191 Australian high school students (see Trompeter et al., 2018 for the detailed study methodology). A passive parental consent procedure was employed, whereby consent was assumed unless parents actively opted their child out of the study. Students provided written assent prior to the study. The current study used data from participants who were students at the 8 schools that participated in the second wave and were aged 13–19 years (*M* = 14.69, *SD* = 1.39), in line with the clinical sample (*n* = 2699). Participants included 1277 boys, 1410 girls, and 12 participants who indicated their gender to be ‘other’. Most participants were born in Australia (83.7%) or Asia (11.4%). 4.9% of participants identified as Aboriginal/Torres Strait Islander.

#### Clinical Sample

 Participants from the clinical sample were part of the TrEAT study, a clinical database of people attending treatment services for an eating disorder. Data were included from seven services located in three major cities in Australia (Sydney and Perth) and New Zealand (Auckland). Before or during their first treatment session, all clients aged 13 years and over with a suspected eating disorder were asked to complete a self-report questionnaire on eating disorder symptoms. Clients were informed about the research study and asked for separate consent for their data to be used for research purposes. Clients aged 16 years or over gave informed consent directly, parents provided consent for their child aged less than 16 years. Permission was granted by the clinics in this study to add a questionnaire on emotion regulation over the period October 2019 to April 2021. For the current study, data of participants aged 13–19 years (*M* = 16.62, *SD* = 1.56), who consented for their data to be used for research purposes (82.9% consent rate), were included (*n* = 149). Participants included 6 boys and 143 girls. Most participants were born in Australia (68.5%), New Zealand (16.8%) or Asia (8.7%). 2% of participants identified as Aboriginal/Torres Strait Islander or New Zealand Māori.

## Measures

### Weight/Shape Concerns

All participants’ weight/shape concerns were assessed using the combined weight and shape concerns subscale of the Eating Disorder Examination Questionnaire (EDE-Q; Fairburn et al., 2008). The measure includes 12 items regarding eating disorder related body image concerns over the previous 28 days. Participants are asked to rate the frequency/severity of their weight and shape concerns (e.g., *How dissatisfied have you been with your shape*) on a 7-point Likert scale (0 = *No days/Not at all* to 6 = *Everyday/Markedly*). Items on the subscale are averaged to provide a mean score from 0 to 6, whereby higher scores indicate higher severity. The subscale has demonstrated good internal consistency among Australian adolescents (Gall et al., 2016; Mond et al., 2014). Excellent internal consistency was shown in the current study both in the community sample (Cronbach’s alpha = 0.96; McDonald's ω = 0.96) and in the clinical sample (Cronbach’s alpha = 0.95; McDonald's ω = 0.96).

### Emotion Dysregulation

To assess participants’ difficulties in emotion regulation the Difficulties in Emotion Regulation Scale – Short Form (DERS-SF) was used (Kaufman et al., 2016). This measure is a short form (18 items) of the original Difficulties in Emotion Regulation Scale (Gratz & Roemer, 2008), a widely used measure of emotion dysregulation. In line with the original scale, the measure examines six factors of emotion dysregulation: non-acceptance of emotional responses, difficulties engaging in goal-directed responses, impulse control difficulties, lack of emotional awareness, lack of emotion regulation strategies, and lack of emotional clarity. An average score measuring participants’ emotion dysregulation was obtained by a mean score of all items, with a range of 1 to 5, whereby higher scores indicate greater difficulties in emotion regulation. The scale has demonstrated good internal consistency and concurrent validity with internalizing symptoms among adolescents (Kaufman et al., 2016). Excellent internal consistency was shown in the current study both in the community sample (Cronbach’s alpha = 0.94; McDonald's ω = 0.92) and in the clinical sample (Cronbach’s alpha = 0.89; McDonald's ω = 0.90).

### Eating Disorder Behaviors

Participants’ disordered eating symptoms were assessed using items from the EDE-Q (Fairburn et al., 2008) to obtain a frequency score for binge eating, purging (combined vomiting and laxative use), and driven exercise. Using an open response format, participants report the frequency of each behavior over the previous 28 days. The response format for the fasting question differed between the two samples. For the clinical sample, the item from the Restraint subscale of the EDE-Q which employs a 7-point Likert type scale (0 = *0 days* to 6 = *Every Day*) was used. Participants in the community sample were asked to respond to an item that was based on the wording of the EDE-Q item for fasting but used an open response format for consistency with the other behavioral items in the broader survey. To ensure consistency, the scores from the community sample were placed in bands corresponding to the 7-point Likert scale used in the clinical sample, as has been done previously (Trompeter et al., 2021b). All disordered eating behaviors were treated as continuous variables, with higher scores indicating a higher frequency of the behavior.

## Data Analytic Plan

Analyses were conducted in a zero-inflated Poisson regression framework using full information maximum likelihood estimation in Mplus version 8 (Muthén & Muthén, 2018), which uses all available data to estimate model parameters. The zero-inflated Poisson regression uses a mixture of a Poisson distribution of count data with an excess of zero counts. Using this regression, the occurrence of the behavior (zero-inflated part) and the frequency of the behavior for those estimated to not represent excess zeros (Poisson part) are examined separately in the same model. Using zero-inflated models is recommended for use with highly skewed clinical data (Gonzalez-Blanks et al., 2020), including eating disorder symptoms (Schaumberg et al., 2018).

Prior to analyses, all independent variables were standardized. Due to the large range of values for binge eating, driven exercise, and purging, values were winsorized to three standard deviations (un-winsorized results are reported in Supplementary 1). To control for multiple comparisons, the Benjamini–Hochberg procedure was used with a paper-wide false discovery rate of 0.05, resulting in a critical alpha of 0.02. All analyses controlled for age and BMI percentile due to their association with eating disorder characteristics (Hay et al., 2015). While we had initially planned to also control for gender, this was not possible due to the small number of male participants in the clinical sample. Supplementary analyses were conducted to examine potential gender differences through multi-group analyses in the associations using the community sample (see Supplementary 2).

To examine the proposed associations, four separate regression models were run, each examining the association between emotion dysregulation, weight/shape concerns, and eating disorder behaviors (fasting, binge eating, purging, driven exercise). Specifically, in the first step each eating disorder behavior (fasting, binge eating, purging, driven exercise) was regressed on the main effect of weight and shape concerns and emotion dysregulation. Multi-group analyses were conducted at this step to examine whether these associations differed by sampling group (clinical vs community). To examine this, all associations were constrained between groups, and the overall difference in fit was tested using a Wald-test, which assesses whether parameters are equal between the two groups. An interaction term between weight and shape concerns and emotion dysregulation was then added to each model. Significant interaction terms were probed using simple slope analysis.

## Results

### Sample Characteristics

As shown in Table 1, the two samples differed significantly in terms of demographics, as well as the variables of interest. Small differences were observed in terms of BMI percentile, binge eating frequency and presence, and fasting presence. Moderate differences were observed in terms of gender and purging frequency and presence. Large differences were observed in terms of age, weight and shape concerns, emotion dysregulation, and fasting frequency. Interestingly, no significant or substantive difference was observed in regards to driven exercise frequency or presence.

**Table 1:** Descriptive statistics of demographic and psychological factors in both samples. Means and standard deviations or percentages as appropriate are presented

Variables	Community sample	Clinical sample	Effect size
	*M* (*SD*)		
Age (in years)	14.69 (1.39)*	16.62 (1.56)*	*d* = 1.31
BMI-percentile	52.36 (30.63)*	39.73 (29.69)*	*d* = 0.42
Weight/shape concerns	1.69 (1.80)*	4.45 (1.58)*	*d* = 1.63
Emotion Dysregulation	2.25 (0.78)*	3.13 (0.76)*	*d* = 1.13
Binge Eating Frequency (past 28 days)	2.79 (6.08)*	6.03 (9.03)*	*d* = 0.42
Purging Frequency (past 28 days)	0.43 (2.48)*	3.62 (7.06)*	*d* = 0.53
Driven Exercise Frequency (past 28 days)	2.89 (6.71)	3.22 (7.05)	*d* = 0.05
Fasting Frequency (past 28 days)	0.40 (0.93)*	1.60 (2.06)*	*d* = 0.75
	*N* (%)		
Gender (% female)	52.2*	96.0*	*V* = 0.41
Binge Eating Presence (% yes)	37.2*	52.3*	*V* = 0.07
Purging Presence (% yes)	9.4*	40.9*	*V* = 0.22
Driven Exercise Presence (% yes)	30.5	29.5	*V* = 0.01
Fasting Presence (% yes)	22.5*	53.7*	*V* = 0.16

Un-winsorized results are reported. Benjamini–Hochberg corrected critical value = 0.02. Significant associations are indicated (*)

## Regression Analysis: Binge Eating

Firstly, the main effects of weight/shape concerns and emotion dysregulation on binge eating were examined, controlling for age and BMI percentile. Multi-group analysis revealed that the two samples were not significantly different (χ^2^(8) = 7.13 *p* = 0.523). Results were therefore interpreted for the combined sample. For completeness, findings by group are reported in Supplementary 3. As can be seen in Table 2, in regards to the probability of binge eating versus not binge eating, both main effects were significant, whereby higher levels of weight/shape concerns, as well as emotion dysregulation, were associated with a higher probability of engaging in binge eating. Concerning frequency, only the main effect of emotion dysregulation was significant, whereby higher levels of emotion dysregulation were associated with more frequent binge eating.

**Table 2:** Regression analysis examining the relationship with binge eating

Variables	Probability of behavior			Frequency of behavior		
	OR	*p*-value	95% CI	B	*p*-value	95% CI
Step 1						
Weight/shape concerns	1.20*	< 0.001	[1.14, 1.26]	0.03	0.233	[-0.02, 0.06]
Emotion dysregulation	1.45*	< 0.001	[1.27, 1.65]	0.13*	0.018	[0.02, 0.24]
Step 2						
Weight/shape concerns X Emotion dysregulation	0.90*	< 0.001	[0.85, -0.95]	0.04	0.061	[-0.00, 0.08]

Benjamini–Hochberg corrected critical value = 0.02. Significant associations are indicated (*). Analysis controlled for age and BMI percentile. *OR* Odds ratio

**Fig. 1:**
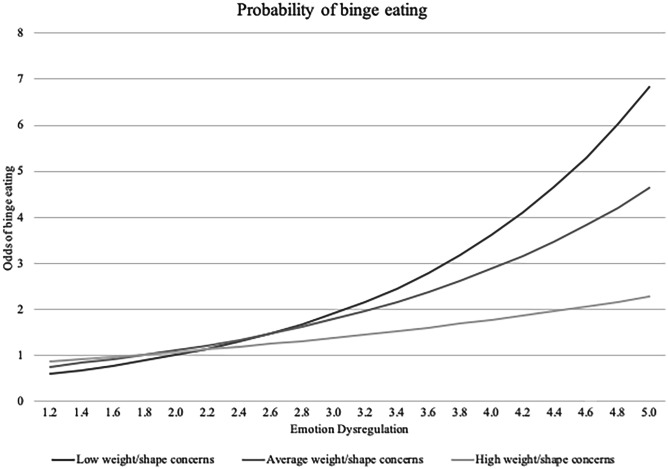
Odds of engaging in binge eating by emotion dysregulation and weight/shape concerns

Secondly, the interaction term between weight/shape concerns and emotion dysregulation was added. The interaction emerged as significant in regards to the probability of engaging in binge eating, but not frequency, whereby weight/shape concerns attenuated the relationship. Simple slopes revealed that the association between emotion dysregulation and probability of binge eating was significant at low levels (OR = 1.89, *p* < 0.001), average levels (OR = 1.55, *p* < 0.001), and high levels (OR = 1.27, *p* = 0.002) of weight/shape concerns (see Fig. 1). Using the Johnson-Neymar technique, we identified the region of non-significance at 1.40 SD of weight and shape concerns, whereby the association between emotion dysregulation and probability of binge eating was no longer significant for adolescents with weight/shape concerns higher than 1.40 SD.

## Regression Analysis: Fasting

Firstly, the main effects of weight/shape concerns and emotion dysregulation on fasting were examined, controlling for age and BMI percentile. Multi-group analysis revealed that the two samples were not significantly different (χ^2^(8) = 7.66, *p* = 0.468). Results were therefore interpreted for the combined sample. For completeness, findings by group are reported in Supplementary 3. As can be seen in Table 3, in regards to the probability of fasting versus not fasting, only weight/shape concerns had a significant main effect, whereby higher levels of weight/shape concerns were associated with a higher probability of fasting. However, concerning frequency, both weight/shape concerns and emotion dysregulation were significant, whereby higher levels of weight/shape concerns and emotion dysregulation were associated with more frequent fasting.

**Table 3:** Regression analysis examining the relationship with fasting

Variables	Probability of behavior			Frequency of behavior		
	OR	*p*-value	95% CI	B	*p*-value	95% CI
Step 1						
Weight/shape concerns	1.57*	< 0.001	[1.41, 1.75]	0.21*	< 0.001	[0.14, 0.27]
Emotion dysregulation	1.26	0.107	[0.95, 1.65]	0.19*	0.009	[0.05, 0.34]
Step 2						
Weight/shape concerns X Emotion dysregulation	0.94	0.410	[0.02, 1.08]	-0.00	0.977	[-0.09, 09]

Benjamini–Hochberg corrected critical value = 0.02. Significant associations are indicated (*). Analysis controlled for age and BMI percentile. *OR* Odds ratio

Secondly, the interaction term between weight/shape concerns and emotion dysregulation was added. The interaction term was not significant for either probability or frequency of fasting.

## Regression Analysis: Purging

Firstly, the main effects of weight/shape concerns and emotion dysregulation on purging were examined, controlling for age and BMI percentile. Multi-group analysis revealed that the two samples were significantly different (χ^2^(8) = 26.17, *p* = 0.001). Results were therefore interpreted separately by group. As can be seen in Table 4, concerning the probability of purging versus not purging, the main effect of weight/shape concerns was significant for both samples, whereby higher weight/shape concerns were associated with a higher probability of purging. Additionally, in the community sample emotion dysregulation was also significant, whereby higher emotion dysregulation was associated with a higher probability of purging. Concerning frequency, neither weight/shape concerns nor emotion dysregulation were significant.

**Table 4:** Regression analysis examining the relationship with purging

	Variables	Probability of behavior				Frequency of behavior		
		OR	*p*-value	95% CI	B		*p*-value	95% CI
Community Sample	Step 1							
	Weight/shape concerns	1.46*	< 0.001	[1.34, 1.58]	0.05		0.184	[-0.03, 0.14]
	Emotion dysregulation	1.58*	< 0.001	[1.32, 1.92]	0.10		0.368	[-0.12, 0.32]
	Step 2							
	Weight/shape concerns X Emotion dysregulation	1.00	0.979	[0.92, 1.08]	-0.01		0.789	[-0.09, 0.07]
Clinical Sample	Step 1							
	Weight/shape concerns	1.92*	< 0.001	[1.49, 2.46]	0.01		0.947	[-0.15, 0.16]
	Emotion dysregulation	0.93	0.780	[0.57, 1.55]	0.15		0.207	[-0.08, 0.38]
	Step 2							
	Weight/shape concerns X Emotion dysregulation	1.09	0.441	[0.87, 1.38]	-0.06		0.475	[-0.22, 0.10]

Benjamini–Hochberg corrected critical value = 0.02. Significant associations are indicated (*). Analysis controlled for age and BMI percentile. *OR* Odds ratio

Secondly, the interaction term between weight/shape concerns and emotion dysregulation was added. The interaction terms were not significant in either sample.

## Regression Analysis: Driven Exercise

Firstly, the main effects of weight/shape concerns and emotion dysregulation on driven exercise were examined, controlling for age and BMI percentile. Multi-group analysis revealed that the two samples were significantly different (χ^2^(8) = 50.91, *p* < 0.001). Results were therefore interpreted separately by group. As can be seen in Table 5, concerning the probability of driven exercise versus no driven exercise, only the main effect of weight/shape concerns was significant in the community sample, whereby higher weight/shape concerns were significantly associated with a higher probability of driven exercise. In regards to frequency, the main effect of weight/shape concerns was significant in the community sample, whereby higher weight/shape concerns were associated with more frequent driven exercise. No associations were significant in the clinical sample.

**Table 5:** Regression analysis examining the relationship with driven exercise

	Variables	Probability of behavior			Frequency of behavior		
		OR	*p*-value	95% CI	B	*p*-value	95% CI
Community Sample	Step 1						
	Weight/shape concerns	1.65*	< 0.001	[1.55, 1.75]	0.13*	< 0.001	[0.08, 0.17]
	Emotion dysregulation	0.99	0.895	[0.85, 1.15]	-0.05	0.435	[-0.18, 0.08]
	Step 2						
	Weight/shape concerns X Emotion dysregulation	0.88*	< 0.001	[0.83, 0.93]	0.01	0.683	[-0.03, 0.05]
Clinical Sample	Step 1						
	Weight/shape concerns	1.08	0.523	[0.85, 1.36]	0.19	0.42	[0.01, 0.36]
	Emotion dysregulation	0.84	0.514	[0.49, 1.42]	-0.27	0.101	[-0.59, 0.05]
	Step 2						
	Weight/shape concerns X Emotion dysregulation	1.01	0.944	[0.84, 1.21]	-0.00	0.995	[-0.18, 0.18]

Benjamini–Hochberg corrected critical value = 0.02. Significant associations are indicated (*). Analysis controlled for age and BMI percentile. *OR* Odds ratio

**Fig. 2:**
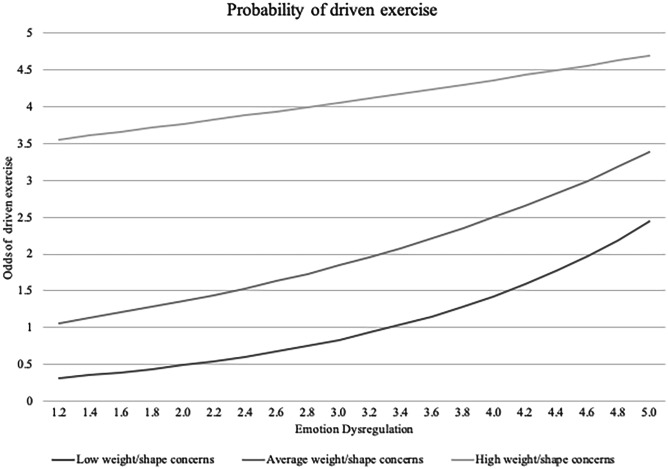
Odds of engaging in driven exercise by emotion dysregulation and weight/shape concerns in the community sample

Secondly, the interaction term between weight/shape concerns and emotion dysregulation was added. This interaction was significant in the community sample in terms of the probability of driven exercise, whereby emotion dysregulation attenuated the relationship (see Fig. 2). Simple slopes revealed that the association between emotion dysregulation and probability of driven exercise was significant at low levels (OR = 1.71, *p* < 0.001), and average levels (OR = 1.36, *p* = 0.003) of weight/shape concerns, but not high levels (OR = 1.08, *p* = 0.320). Using the Johnson-Neymar technique, we identified the region of insignificance at 0.56 SD of weight and shape concerns.

## Supplementary Analysis: Gender Difference

Supplementary analyses were conducted to examine potential gender differences through multi-group analyses in the associations using the community sample (see Supplementary 2). Few gender differences were found regarding the main associations. However, significant gender non-invariance in the parameter for the associations between probability of purging and weight/shape concerns was found. Examination of confidence intervals in the split sample showed a stronger association among girls compared to boys. Similarly, significant gender non-invariance in the parameter for the associations between emotion dysregulation and probability of engaging in driven exercise was found. Examination of confidence intervals revealed that among boys, emotion dysregulation was associated with higher probability of engaging in driven exercise, while for girls emotion dysregulation was associated with lower probability of engaging in driven exercise for girls.

## Discussion

The current study examined whether emotion dysregulation was associated with eating disorder symptoms and whether weight and shape concerns would moderate these associations among adolescents. Findings suggest that rather than having a multiplicative effect, weight and shape concerns and emotion dysregulation each had unique, additive associations with the probability and frequency of several eating disorder behaviors. To account for adolescents not engaging in certain eating disorder behaviors, the current study distinguished between probability of engaging in eating disorder behaviors, whereby adolescents who reported certain eating disorder behaviors were compared with those not reporting such a behavior, and frequency, whereby only adolescents who reported engaging in certain eating disorder behaviours were considered.

In terms of the probability of engaging in eating disorder behaviors, emotion dysregulation was found to have a unique positive association with engaging in binge eating and purging in the community sample. Weight and shape concerns were also found to have a unique association with engaging in binge eating, fasting, and purging in both samples, as well as driven exercise in the community sample. These findings are in line with the CBT-E model, whereby weight and shape concerns are a core factor of all eating disorder behaviors, whereas emotion dysregulation is linked only with binge eating and purging (Fairburn et al., 2003).

Regarding the frequency of eating disorder behaviors, different patterns emerged. Emotion dysregulation was found to have unique associations with frequency of binge eating and fasting. Weight and shape concerns were also found to have unique associations with frequency of fasting and driven exercise in the community sample. These findings highlight the proposed regulatory nature of both binge eating and fasting (Engel et al., 2013; Svaldi et al., 2019) and underscore the importance of emotion dysregulation in binge eating pathology (Leehr et al., 2015).

A major aim of the current study was to examine the interplay between emotion dysregulation and weight and shape concerns in regards to eating disorder behaviors. Results showed that weight and shape concerns moderated the association between emotion dysregulation and the probability of engaging in binge eating and driven exercise. However, the relationship was such that the strongest association between emotion dysregulation and these behaviors was observed among adolescents with the lowest levels of weight and shape concerns. These findings conflicted with our hypothesis. One potential explanation for these unexpected findings may be related to a ceiling effect. If adolescents already experience high levels of weight and shape concerns, there may be limited additional ‘risk’ from also experiencing high levels of emotion dysregulation. However, for adolescents with lower levels of weight and shape concerns, emotion dysregulation may be amplifying the likelihood of adolescents engage in eating disorder behaviours. This hypothesis would be interesting to explore in future longitudinal research.

While the current study had several strengths, including the use of both a clinical and community sample of adolescents, several limitations should also be considered. Firstly, the current study relied on cross-sectional data. Thus, no inferences about the direction of the associations can be made, precluding any inferences about whether emotion dysregulation is truly involved in the onset or maintenance of eating disorder behaviors. Further, non-significant results from the clinical sample should be interpreted with caution. Given the small sample size and the power required to detect unique effects, as well as interaction effects (Blake & Gangestad, 2020), it is likely that we were unable to detect potentially important associations in this group. Lastly, due to the low frequency of male participants in the clinical sample, we were unable to control for or assess the impact of gender on the investigated associations. Supplementary analyses using the community sample pointed towards gender differences in the association between driven exercise and emotion dysregulation. Future research should consider potential gender differences in these associations in particular.

The findings from the current study have important implications for both prevention and intervention programs aimed at reducing eating disorder behaviors among adolescents, as well as current theoretical models. Firstly, the current study suggests that in addition to the well-established disorder-specific risk factor of weight and shape concerns, emotion dysregulation may play an important part in both the probability of engaging in and the frequency of certain eating disorder behaviors. As such, treatments for eating disorders may benefit from integrating emotion regulation-based approaches, such as dialectic behavioral therapy (see Reilly et al., 2020 for review), especially when treating adolescents who engage in binge eating and/or fasting. Similarly, prevention programs may benefit from teaching adolescents emotion regulation skills more broadly. This may be best done at the universal rather than targeted prevention level, as even at low and moderate levels of weight and shape concerns, emotion dysregulation was associated with higher odds of engaging in disordered eating.

Secondly, the current study has important theoretical implications. The current version of the CBT-E model suggests that ‘mood intolerance’ (a facet of emotion dysregulation) maintains both binge eating and purging behaviors, but not dietary restraint or other weight-control behaviors (Fairburn et al., 2003). However, we have previously theorized that emotion dysregulation is associated with all eating behaviors represented in the CBT-E model (Trompeter et al., 2021a). The current study provides partial support for these suggestions, by firstly showing that emotion dysregulation is a key factor in eating disorder behaviors. Specifically, emotion dysregulation showed unique associations here with fasting, purging, and binge eating, after controlling for concurrent weight and shape concerns, age, and BMI percentile. Future research should expand these findings to also examine other weight-control behaviors, such as dietary restraint, to further our understanding of these associations. Additionally, the current study did not find the proposed interactions between weight and shape concerns and emotion dysregulation and eating disorder behaviors. This may have, in part, been due to the small sample size, and hence low power, in the clinical sample. Future studies should examine these potential interactions in a larger clinical sample of adolescents.

In conclusion, the current study adds to a growing body of literature suggesting that emotion dysregulation is a key transdiagnostic factor of eating disorder behaviors among adolescents. By investigating the relationship between emotion dysregulation and both the probability of eating disorder behaviors occurring as well as the frequency of these behaviors, we were able to provide preliminary insights into the potential role of emotion dysregulation in these behaviors—both independently and in conjunction with weight and shape concerns.

## Supplementary Information

Below is the link to the electronic supplementary material.

Supplementary file1 (DOCX 20 KB)

Supplementary file2 (DOCX 21 KB)

Supplementary file3 (DOCX 30 KB)

## Data Availability

Deidentified data are available upon request from the senior author (D.M.), pertaining to approval from the authors’ institutional ethics committee.

## Code Availability

All code is available: https://osf.io/69pk8/?view_only=5938a76bb1184b878712b07d35fa088a
