# Supplementary material for: Emotion Dysregulation and Eating Disorder Symptoms: Examining Distinct Associations and Interactions in Adolescents
Source: Res Child Adolesc Psychopathol. 2022 Jan 14;50(5):683–94. doi: 10.1007/s10802-022-00898-1 (PMC9054869; doi:10.1007/s10802-022-00898-1)
Supplement: Supplementary file 1 — Supplementary file1 (DOCX 20 KB) [file 10802_2022_898_MOESM1_ESM.docx]

**Supplementary material: Results by group**

**Binge eating**

Table S1. Regression analysis examining the relationship with binge eating group.

|  | Variables | Probability of behavior | | | | Frequency of behavior | | |
| --- | --- | --- | --- | --- | --- | --- | --- | --- |
|  |  | OR | *p*-value | 95% CI | B | | *p*-value | 95% CI |
| Community | Step 1 |  |  |  |  | |  |  |
|  | Weight/shape concerns | 1.22* | <.001 | [1.15, 1.28] | .02 | | .481 | [-.03, .06] |
|  | Emotion dysregulation | 1.43* | <.001 | [1.26, 1.66] | .12 | | .040 | [.01, .24] |
|  | Step 2 |  |  |  |  | |  |  |
|  | Weight/shape concerns  X  Emotion dysregulation | 0.90* | <.001 | [0.85, 0.95] | .03 | | .134 | [-.01, .08] |
| Clinical | Step 1 |  |  |  |  | |  |  |
|  | Weight/shape concerns | 0.96 | .750 | [0.77, 1.20] | .04 | | .641 | [-.12, .19] |
|  | Emotion dysregulation | 1.73* | <.001 | [1.04, 2.90] | .11 | | .358 | [-.13, .36] |
|  | Step 2 |  |  |  |  | |  |  |
|  | Weight/shape concerns  X  Emotion dysregulation | 0.77 | .058 | [0.59, 1.01] | .10 | | .222 | [-.06, .25] |

*Note.* Benjamini-Hochberg corrected critical value = 0.02. Significant associations are indicated (*). Analysis controlled for age and BMI percentile. OR = Odds ratio

**Fasting**

Table S2. Regression analysis examining the relationship with fasting by group.

|  | Variables | Probability of behavior | | | | Frequency of behavior | | |
| --- | --- | --- | --- | --- | --- | --- | --- | --- |
|  |  | OR | *p*-value | 95% CI | B | | *p*-value | 95% CI |
| Community | Step 1 |  |  |  |  | |  |  |
|  | Weight/shape concerns | 1.60* | <.001 | [1.41, 1.81] | .19* | | <.001 | [.11, .26] |
|  | Emotion dysregulation | 1.38 | .055 | [0.99, 1.92] | .16 | | .064 | [-.01, .33] |
|  | Step 2 |  |  |  |  | |  |  |
|  | Weight/shape concerns  X  Emotion dysregulation | 0.98 | .818 | [0.79, 1.20] | -.03 | | .629 | [-.14, .08] |
| Clinical | Step 1 |  |  |  |  | |  |  |
|  | Weight/shape concerns | 1.56* | .009 | [1.12, 2.18] | .27* | | .013 | [.06, .49] |
|  | Emotion dysregulation | 0.89 | .784 | [0.40, 1.99] | .21 | | .224 | [-.13, .58] |
|  | Step 2 |  |  |  |  | |  |  |
|  | Weight/shape concerns  X  Emotion dysregulation | 1.35 | .286 | [0.78, 2.34] | -.06 | | .726 | [-.37, .26] |

*Note.* Benjamini-Hochberg corrected critical value = 0.02. Significant associations are indicated (*). Analysis controlled for age and BMI percentile. OR = Odds ratio
