# Supplementary material for: Emotion Dysregulation and Eating Disorder Symptoms: Examining Distinct Associations and Interactions in Adolescents
Source: Res Child Adolesc Psychopathol. 2022 Jan 14;50(5):683–94. doi: 10.1007/s10802-022-00898-1 (PMC9054869; doi:10.1007/s10802-022-00898-1)
Supplement: Supplementary file 2 — Supplementary file2 (DOCX 21 KB) [file 10802_2022_898_MOESM2_ESM.docx]

**Supplementary 1: Un-winsorized results**

Table S1. Regression analysis examining the relationship with binge eating (un-winsorized)

| Variables | Probability of behavior | | | Frequency of behavior | | |
| --- | --- | --- | --- | --- | --- | --- |
|  | OR | *p*-value | 95% CI | B | *p*-value | 95% CI |
| Step 1 |  |  |  |  |  |  |
| Weight/shape concerns | 1.20* | <.001 | [1.14, 1.26] | .01 | .700 | [-.04, .05] |
| Emotion dysregulation | 1.44* | <.001 | [1.27, 1.63] | .16* | .012 | [.03, .28] |
| Step 2 |  |  |  |  |  |  |
| Weight/shape concerns  X  Emotion dysregulation | 0.90* | <.001 | [0.85, 0.95] | .05 | .023 | [.01, .10] |

*Note.* Benjamini-Hochberg corrected critical value = 0.02. Significant associations are indicated (*). Analysis controlled for age and BMI percentile. OR = Odds ratio

Table S2. Regression analysis examining the relationship with purging (un-winsorized).

|  | Variables | Probability of behavior | | | Frequency of behavior | | |
| --- | --- | --- | --- | --- | --- | --- | --- |
|  |  | OR | *p*-value | 95% CI | B | *p*-value | 95% CI |
| Community Sample | Step 1 |  |  |  |  |  |  |
|  | Weight/shape concerns | 1.47* | <.001 | [1.35, 1.58] | .05 | .406 | [-.06, .15] |
|  | Emotion dysregulation | 1.51* | <.001 | [1.24, 1.85] | .26 | .044 | [.01, .52] |
|  | Step 2 |  |  |  |  |  |  |
|  | Weight/shape concerns  X  Emotion dysregulation | 1.00 | .975 | [0.91, 1.08] | -.03 | .679 | [-.14, .09] |
| Clinical Sample | Step 1 |  |  |  |  |  |  |
|  | Weight/shape concerns | 1.93* | <.001 | [1.51, 2.46] | .09 | .407 | [-.12, .29] |
|  | Emotion dysregulation | 0.93 | .779 | [0.56, 1.55] | .42* | .017 | [.08, .77] |
|  | Step 2 |  |  |  |  |  |  |
|  | Weight/shape concerns  X  Emotion dysregulation | 1.10 | .412 | [0.88, 1.38] | .02 | .875 | [-.26, .29] |

*Note.* Benjamini-Hochberg corrected critical value = 0.02. Significant associations are indicated (*). Analysis controlled for age and BMI percentile. OR = Odds ratio

Table S3. Regression analysis examining the relationship with driven exercise (un-winsorized)

|  | Variables | Probability of behavior | | | Frequency of behavior | | |
| --- | --- | --- | --- | --- | --- | --- | --- |
|  |  | OR | *p*-value | 95% CI | B | *p*-value | 95% CI |
| Community Sample | Step 1 |  |  |  |  |  |  |
|  | Weight/shape concerns | 1.65* | <.001 | [1.55, 1.75] | .12* | <.001 | [.07, .19] |
|  | Emotion dysregulation | 0.99 | .899 | [0.87, 1.17] | -.04 | .605 | [-.18, .11] |
|  | Step 2 |  |  |  |  |  |  |
|  | Weight/shape concerns  X  Emotion dysregulation | 0.88* | <.001 | [0.83, 0.94] | .02 | .543 | [-.04, .07] |
| Clinical Sample | Step 1 |  |  |  |  |  |  |
|  | Weight/shape concerns | 1.08 | .522 | [0.74, 1.17] | .20 | .043 | [.01, .39] |
|  | Emotion dysregulation | 0.84 | .513 | [0.70, 2.04] | -.23 | .199 | [-.59, .12] |
|  | Step 2 |  |  |  |  |  |  |
|  | Weight/shape concerns  X  Emotion dysregulation | 1.01 | .942 | [0.84, 1.21] | .02 | .827 | [-.17, .21] |

*Note.* Benjamini-Hochberg corrected critical value = 0.02. Significant associations are indicated (*). Analysis controlled for age and BMI percentile. OR = Odds ratio
