# Supplementary material for: Emotion Dysregulation and Eating Disorder Symptoms: Examining Distinct Associations and Interactions in Adolescents
Source: Res Child Adolesc Psychopathol. 2022 Jan 14;50(5):683–94. doi: 10.1007/s10802-022-00898-1 (PMC9054869; doi:10.1007/s10802-022-00898-1)
Supplement: Supplementary file 3 — Supplementary file3 (DOCX 30 KB) [file 10802_2022_898_MOESM3_ESM.docx]

**Supplementary analysis**

**Gender comparisons: Binge eating**

In regards to binge eating, a significant gender difference was detected. Multi-group analysis revealed that overall the associations were significantly different between boys and girls (χ^2^(8) = 24.06, *p* = .002). However, all confidence intervals for the main effects overlapped, indicating that there were no significant differences in these associations between boys and girls. As such, multi-group analyses were re-run, isolating one association to determine the source of the difference. Findings showed that the only association showing significant gender differences were the association between probability of binge eating and BMI percentile (χ^2^(1) = 12.61, *p* < .001).

Results from the regression model split by gender are shown in Table S1 below.

Table S1. Regression analysis examining the relationship with binge eating in the community sample by gender.

|  | Variables | Probability of behavior | | | | Frequency of behavior | | |
| --- | --- | --- | --- | --- | --- | --- | --- | --- |
|  |  | OR | *p*-value | 95% CI | B | | *p*-value | 95% CI |
| Boys | Step 1 |  |  |  |  | |  |  |
|  | Weight/shape concerns | 1.30* | <.001 | [1.17, 1.44] | -.01 | | .822 | [-.09, .07] |
|  | Emotion dysregulation | 1.55* | <.001 | [1.25, 1.93] | .09 | | .327 | [-.10, .28] |
|  | Step 2 |  |  |  |  | |  |  |
|  | Weight/shape concerns  X  Emotion dysregulation | 0.90 | .070 | [0.80, 1.01] | .02 | | .532 | [-.05, .09] |
| Girls | Step 1 |  |  |  |  | |  |  |
|  | Weight/shape concerns | 1.25* | <.001 | [1.17, 1.34] | .07 | | .043 | [.00, .13] |
|  | Emotion dysregulation | 1.36* | <.001 | [1.15, 1.61] | .17 | | .032 | [.02, .33] |
|  | Step 2 |  |  |  |  | |  |  |
|  | Weight/shape concerns  X  Emotion dysregulation | 0.88 | .002 | [0.82, 0.96] | .01 | | .846 | [-.06, .08] |

*Note.* Benjamini-Hochberg corrected critical value = 0.02. Significant associations are indicated (*). Analysis controlled for age and BMI percentile. OR = Odds ratio

**Gender comparisons: Fasting**

Regarding fasting, no significant gender difference was detected. Multi-group analysis revealed that overall the associations were not significantly different between boys and girls (χ^2^(8) = 15.01 *p* = .050). Results from the regression model split by gender are shown in Table S2 below.

Table S2. Regression analysis examining the relationship with fasting in the community sample by gender.

|  | Variables | Probability of behavior | | | | Frequency of behavior | | |
| --- | --- | --- | --- | --- | --- | --- | --- | --- |
|  |  | OR | *p*-value | 95% CI | B | | *p*-value | 95% CI |
| Boys | Step 1 |  |  |  |  | |  |  |
|  | Weight/shape concerns | 1.76* | .002 | [1.24, 2.52] | .16 | | .040 | [.01, .31] |
|  | Emotion dysregulation | 1.45 | .191 | [0.83, 2.52] | .14 | | .385 | [-.18, .46] |
|  | Step 2 |  |  |  |  | |  |  |
|  | Weight/shape concerns  X  Emotion dysregulation | 1.24 | .643 | [0.50, 3.09] | -.09 | | .423 | [-.31, .13] |
| Girls | Step 1 |  |  |  |  | |  |  |
|  | Weight/shape concerns | 1.69* | <.001 | [1.43, 2.01] | .22* | | <.001 | [.12, .33] |
|  | Emotion dysregulation | 1.26 | .316 | [0.80, 1.98] | .19 | | .107 | [-.04, .42] |
|  | Step 2 |  |  |  |  | |  |  |
|  | Weight/shape concerns  X  Emotion dysregulation | 0.93 | .557 | [0.73, 1.19] | -.01 | | .924 | [-.15, .14] |

*Note.* Benjamini-Hochberg corrected critical value = 0.02. Significant associations are indicated (*). Analysis controlled for age and BMI percentile. OR = Odds ratio

**Gender comparisons: Purging**

Regarding purging, a significant gender difference was detected. Multi-group analysis revealed that overall the associations were significantly different between boys and girls (χ^2^(8) = 23.76, *p* = .003). Inspection of the confidence intervals for the main effects indicated that the associations between probability of purging and weight/shape concerns was stronger among girls compared to boys. Results from the regression model split by gender are shown in Table S3 below.

Table S3. Regression analysis examining the relationship with purging in the community sample by gender.

|  | Variables | Probability of behavior | | | | Frequency of behavior | | |
| --- | --- | --- | --- | --- | --- | --- | --- | --- |
|  |  | OR | *p*-value | 95% CI | B | | *p*-value | 95% CI |
| Boys | Step 1 |  |  |  |  | |  |  |
|  | Weight/shape concerns | 1.31* | <.001 | [1.14, 1.51] | .10 | | .062 | [-.01, .21] |
|  | Emotion dysregulation | 2.00* | <.001 | [1.46, 2.70] | .04 | | .767 | [-.23, .31] |
|  | Step 2 |  |  |  |  | |  |  |
|  | Weight/shape concerns  X  Emotion dysregulation | 1.02 | .843 | [0.88, 1.18] | -.01 | | .920 | [-.12, .11] |
| Girls | Step 1 |  |  |  |  | |  |  |
|  | Weight/shape concerns | 1.73* | <.001 | [1.53, 1.95] | .22* | | .020 | [.03, .40] |
|  | Emotion dysregulation | 1.33 | .025 | [1.04, 1.70] | .10 | | .567 | [-.25, .46] |
|  | Step 2 |  |  |  |  | |  |  |
|  | Weight/shape concerns  X  Emotion dysregulation | 1.02 | .772 | [0.88, 1.18] | -.08 | | .322 | [-.24, .08] |

*Note.* Benjamini-Hochberg corrected critical value = 0.02. Significant associations are indicated (*). Analysis controlled for age and BMI percentile. OR = Odds ratio

**Gender comparisons: Driven exercise**

Regarding driven exercise, a significant gender difference was detected. Multi-group analysis revealed that overall the associations were significantly different between boys and girls (χ^2^(8) = 51.71, *p* < .001). Inspection of the confidence intervals for the main effects indicated that while emotion dysregulation was associated with higher probability of engaging in driven exercise among boys, it was associated with lower probability of engaging in driven exercise for girls. Results from the regression model split by gender are shown in Table S4 below.

Table S4. Regression analysis examining the relationship with driven exercise in the community sample by gender.

|  | Variables | Probability of behavior | | | | Frequency of behavior | | |
| --- | --- | --- | --- | --- | --- | --- | --- | --- |
|  |  | OR | *p*-value | 95% CI | B | | *p*-value | 95% CI |
| Boys | Step 1 |  |  |  |  | |  |  |
|  | Weight/shape concerns | 1.61* | <.001 | [1.44, 1.81] | .15* | | <.001 | [.08, .22] |
|  | Emotion dysregulation | 1.43* | .002 | [1.14, 1.80] | -.25* | | .002 | [-.41, -.09] |
|  | Step 2 |  |  |  |  | |  |  |
|  | Weight/shape concerns  X  Emotion dysregulation | 0.91 | .155 | [0.95, 1.03] | -.02 | | .540 | [-.10, .05] |
| Girls | Step 1 |  |  |  |  | |  |  |
|  | Weight/shape concerns | 1.85* | <.001 | [1.69, 2.02] | .15* | | <.001 | [.09, .21] |
|  | Emotion dysregulation | 0.76* | .006 | [0.62, 0.92] | .07 | | .393 | [-.09, .23] |
|  | Step 2 |  |  |  |  | |  |  |
|  | Weight/shape concerns  X  Emotion dysregulation | 0.90* | .015 | [0.82, 0.98] | -.02 | | .585 | [-.08, .05] |

*Note.* Benjamini-Hochberg corrected critical value = 0.02. Significant associations are indicated (*). Analysis controlled for age and BMI percentile. OR = Odds ratio
